# Metformin Ameliorates Testicular Damage in Male Mice with Streptozotocin-Induced Type 1 Diabetes through the PK2/PKR Pathway

**DOI:** 10.1155/2019/5681701

**Published:** 2019-11-28

**Authors:** Yuning Liu, Zhen Yang, Dongbo Kong, Youzhi Zhang, Wei Yu, Wenliang Zha

**Affiliations:** ^1^Department of Surgery, Clinic Medical College, Hubei University of Science and Technology, Xianning, Hubei 437100, China; ^2^Department of Pharmacology, School of Pharmacy, Hubei University of Science and Technology, Xianning, Hubei 437100, China; ^3^Department of Urology, Xianning Central Hospital, Xianning 437100, China; ^4^Hubei Province Key Laboratory on Cardiovascular, Cerebrovascular, and Metabolic Disorders, Hubei University of Science and Technology, Xianning 437100, China; ^5^National Demonstration Center for Experimental General Medicine Education (Hubei University of Science and Technology), Xianning 437100, China

## Abstract

Approximately 90% of male diabetes mellitus patients have varying degrees of testicular dysfunction. The molecular mechanism underlying diabetes-induced testicular damage has not been thoroughly elucidated. In this research, we sought to determine the influence of metformin (Met) on diabetes-induced testicular injury and the mechanism involved with a focus on testicular dysfunction, apoptosis, autophagy, and prokineticin 2 (PK2) signalling. In our study, C57BL/6J mice were randomly divided into the normal control group, the diabetes group, and the Met-treated group. Streptozotocin (50 mg·kg^−1^·d^−1^) was injected intraperitoneally into the mice for 5 days in a row to induce type 1 diabetes, which was diagnosed by a blood glucose level ≥ 16.7 mmol/L after 7 days. The experimental animals were orally administered Met (250 mg·kg^−1^·d^−1^) for 16 weeks. Properties of testicular function, including sperm motility and the total concentration of epididymal sperm, were assessed. Changes in testicular structure, such as the blood-testis barrier, histological pathology, and organelles, were observed. The levels of apoptosis and expression of related proteins, such as Bax and Bcl-2, were measured. Moreover, autophagy-related proteins, including Beclin-1, p62, and LC3B, as well as the PK2/PKR pathway, which consists of PK2, PKR1, PKR2, AKT, and GSK3*β*, were analysed. Upon the induction of diabetes, reproductive capacity was significantly impaired and a disordered arrangement of testicular seminiferous tubules and destroyed organelles in spermatogenic cells was observed. Met administration preserved testicular function and structure. In addition, in mice with diabetes, the levels of PK2, PKR2, p-Akt, and p-GSK3*β* were significantly decreased at different times, while that of PKR1 was markedly increased, and these changes were normalized by Met. Furthermore, diabetic mice showed increased apoptosis and decreased autophagy in the testes, the effects of which were nullified by Met. These results suggest that Met rescues diabetes-induced testicular damage by attenuating apoptosis and inducing autophagy. This effect is likely mediated by the PK2/PKR/AKT/GSK3*β* signalling pathway.

## 1. Introduction

Diabetes mellitus is a disorder of glucose metabolism caused by an absolute or relative insufficiency of insulin secretion. According to a prediction by the International Diabetes Federation [1], the number of diabetic patients will increase to 693 million in 2045, accounting for approximately 10% of the global population. Recently, researchers have elucidated that testicular dysfunction occurs in a startling number of diabetes cases and that testicular dysfunction has become a prevalent complication of diabetes. Approximately 94.4% of diabetes cases are associated with hypotestosteronaemia, which can lead to diabetic erectile dysfunction [2], and the incidence of sexual and reproductive dysfunction in diabetic patients is 5-10 times higher than that in nondiabetic patients [3, 4]. The major pathological manifestations of diabetes-induced testicular dysfunction include low testosterone levels and compromised reproductive function, which may be attributed to apoptosis and autophagy and may engender hypogonadism. Nevertheless, the precise molecular mechanism by which testicular dysfunction is caused by diabetes remains unclear, and no specific medicines are available for treatment.

Currently, numerous medicines are available for diabetes mellitus, and metformin (Met) is an effective hypoglycaemic drug that can effectively control blood glucose by reducing the absorption of sugar in the intestinal tract, enhancing the intracellular transport of glucose, and inhibiting the production of glycogen in the liver. During its more than 60 years of clinical application, Met has been used not only to control blood glucose levels in patients with diabetes but also to prevent diabetes complications. It has a curative effect on diabetic cardiomyopathy [5] and diabetic retinopathy [6]. Although Met has some effect on other complications caused by diabetes mellitus, it has not been reported for the treatment of testicular dysfunction and the mechanisms of Met in testicular tissue remain unexplored.

Prokineticin 2 (PK2), also called Bombina variegata 8 (Bv8), is a secreted low-molecular-weight protein extracted from the skin secretions of toads [7]. Previous studies have demonstrated that PK2 plays a role in a variety of biological processes, including nerve growth, angiogenesis, the immune response, and inflammation [8–10]. In addition, there is increasing evidence that PK2 plays a role in regulating gonadotropin-releasing hormone (GnRH) for gonadal development, as knockout of the PK2 gene was delayed and deformed in the testes of male mice, leading to spermatogenesis deficiency [8, 11, 12]. Prokineticin receptors (PKRs) are specific G protein-coupled receptors (GPCRs) that comprise two functional complexes, namely, PKR1 and PKR2, which are responsible for the biological effects induced by PK2 in effector cells. Previous studies have shown that PKR2 is more important in the regulation of testicular growth than PKR1 [13]. Although PK2/PKRs play an indispensable role in the reproductive system, there are no reports about their role and mechanism in reproductive injury caused by diabetes mellitus. Accordingly, this study attempted to evaluate the role of Met in diabetes-induced testicular damage, reproductive dysfunction, apoptosis, autophagy, and the PK2/PKR pathway.

## 2. Materials and Methods

### 2.1. Experimental Animals

SPF C57BL/6J male mice (18-22 g, 5-6 weeks) were obtained from the Hubei Laboratory Animal Research Center and housed at a temperature of 22 ± 2°C and a moisture content of 40% under a 12 h light/dark cycle. All experimental subjects were treated according to the Guide for the Care and Use of Laboratory Animals published by the US National Institutes of Health (NIH publication no. 85–23, revised 1996). All experimental procedures were approved by the Institutional Animal Care and Use Committee of Hubei University of Science and Technology.

### 2.2. Induction of Experimental Diabetes

A week after adaptive feeding, mice were randomly divided into 2 groups: the control group (*n* = 60) and the diabetes group (DM, *n* = 100). The DM mice were intraperitoneally injected with streptozotocin (STZ, 50 mg·kg^−1^·d^−1^ in 0.1 mol/L citric acid buffer, pH 4.5) for 5 days in a row [14]. The mice in the control group were injected with an equal amount of citric acid-sodium citrate buffer solution. Random blood glucose was monitored within seven days after STZ injection; the experimental animals with a blood glucose level ≥ 16.7 mmol/L were diagnosed as diabetic, and their blood glucose was monitored weekly [14]. At appropriate time points (2 months, 3 months, 4 months, and 5 months) after the diagnosis of diabetes, the mice were euthanized under anaesthesia, and the testes were quickly removed and stored in Bouin's fixative fluid for histomorphological examination. The other samples were stored at -80°C for other experiments. The epididymis was collected to determine reproductive function.

### 2.3. Treatment with Met

Mice were randomly divided into the normal control group (*n* = 20), the DM group (*n* = 25), and the Met treatment group (DM+Met, *n* = 25). The diabetes model was established according to a previously described method. In the Met treatment group, mice that were diagnosed with diabetes after STZ injection were orally administered Met hydrochloride solution (250 mg·kg^−1^·d^−1^) in potable water for 4 months. Mice in the other groups were administered an equal volume of water.

### 2.4. Biotin Tracer Studies

The skin and fascia of anaesthetized mice were incised to expose the testicular tissue. A total of 50 *μ*L of EZ-link Sulfo-NHS-LC-Biotin (50 mg/mL) was injected under the tunica albuginea using a microinjection needle. Thirty minutes after the skin was sutured, the mice were sacrificed, and their testicles were removed and fixed in a fixative solution. The tissue was embedded in paraffin overnight and sectioned. The tissues were dewaxed in xylene and hydrated in graded ethanol solutions. Antigen retrieval was performed by incubating the sections in 5% skim milk in PBS containing 0.01% Triton X-100 for 15 min. An Alexa Fluor 568-conjugated streptavidin solution was diluted with PBS at a 1 : 200 ratio and added to the sections. The sections were incubated at 37°C for 2 h in a dark room and then washed 3 times with PBS. The sections were incubated with DAPI-containing antifluorescence quenching tablets and observed under a fluorescence microscope.

### 2.5. Measurement of Reproductive Capacity

The cauda epididymes of the mice were placed in physiological saline at 37°C and homogenized. The numbers of active and inactive sperm in 5 visual fields were counted, and the sperm activity rate (active sperm number/total sperm number × 100%) was calculated. The sperm suspension was placed in physiological saline at 37°C and dried on a cell counting plate after blending. The concentration of sperm in the five central squares was counted, and the average was multiplied by 10^9^ to obtain the number of sperm per litre of semen, as previously described [15].

### 2.6. Histopathological Examination

Testicular tissue was soaked in Bouin's fixative solution for 12 h, embedded in paraffin, and sectioned into 4 *μ*m thick slices. After HE staining, changes in the tissue structure were observed under a light microscope. The testicular tissue was immobilized with an electron microscopy fixative and embedded with an acetone-812 embedding agent. The organelle structure of the testicular tissue was observed by transmission electron microscopy.

### 2.7. Terminal Deoxynucleotidyl Transferase-Mediated dUTP Nick End-Labelling (TUNEL) Assay

A TUNEL assay was performed according to the instructions of the TUNEL kit (Roche Applied Science, USA). Paraffin sections were dewaxed in xylene, rehydrated in a 100%~70% ethanol gradient, and sequentially incubated in 10 *μ*g/mL proteinase K at 37°C for 30 min for antigen retrieval, in 1% Triton X-100 at room temperature for 20 min, in TDT-enzyme and fluorescein-labelled dUTP in proportion at 37°C for 2 h, in 10% serum for 30 min at room temperature, and, finally, in a 10% hydrogen peroxide-methanol solution for 15 min. After 30 min of incubation with converter-POD (HRP-labelled fluorescein antibody), TUNEL-positive cells (green under a microscope) were randomly observed under a fluorescence microscope (Leica, USA). The sections were washed three times with PBS after each step.

### 2.8. Immunohistochemical Determination of Protein Expression

The samples were incubated with polyclonal primary antibodies at 4°C for 12 h, washed three times with PBS, and then incubated with a biotinylated horse antimouse immunoglobulin (IgG) solution for 1 h. The samples were rinsed with PBS three more times and incubated with freshly prepared 3,3′-diaminobenzidine (DAB) solution for colour development. Finally, the sections were sealed with neutral resin and observed under an optical microscope.

### 2.9. Real-Time Reverse Transcription Polymerase Chain Reaction (RT-qPCR)

TRIzol reagent was used to extract total RNA from the testicular tissues, and 1 *μ*L of RNA from each sample was reverse transcribed into cDNA with the following PCR programme: 95°C for 10 min followed by 40 cycles of 95°C for 15 s and annealing at 60°C for 60 s. After the reaction was completed, the reaction product was separated by agarose gel electrophoresis, and the results were analysed by the *ΔΔ*CT method.  Table 1 provides the nucleotide sequences of the primers used in this research.

### 2.10. Western Blot Analysis

The testicular tissues were weighed, and approximately 50 mg of tissue was added to RIPA buffer (Cell Signaling Technology, USA) that included protease inhibitors and phosphatase inhibitors. The protein concentration was determined by the BCA protein assay (Beyotime, China). Equivalent amounts of protein were loaded onto gels and separated by SDS-PAGE. Then, the following antibodies were used for Western blotting: Beclin-1, LC3B, protein kinase B (AKT), phosphor-AKT, glycogen synthase kinase-3beta (GSK3*β*), phosphor-GSK3*β*, Bax, Bcl-2 (1 : 1000, Cell Signaling Technology, USA), p62 (1 : 500, Wanleibio, CN), PK2 (1 : 1000, Abcam, USA), and PKR1 and PKR2 (1 : 2000, Santa Cruz Biotechnology, USA). The samples were incubated with the appropriate secondary antibodies for 1 h at room temperature. The blots were analysed with an ECL kit (Meilunbio, CN).

### 2.11. Statistical Analysis

The data are presented as the mean ± SEM of replicated experiments. Analysis was performed by one-way analysis of variance (ANOVA). Differences with *P* values < 0.05 were considered statistically significant.

## 3. Results

### 3.1. General State and Reproductive Potential of Mice over Time

The diabetic mice showed the typical symptoms of diabetes, specifically polydipsia, polyuria, polyphagia, and weight loss. As demonstrated in Table 2, compared with the control group, the diabetic mice had dull, rough, and disorderly hair. In the DM group, body weight was significantly reduced, and a hyperglycaemic status was maintained for a long time (Table 2). In terms of reproductive function, although the total sperm count in the testes of mice in the early stage of diabetes was low compared to that in testes of mice in the normal control group, it was still at a sufficient level to maintain vitality. As the disease progressed, the reproductive capacity of the diabetic mice gradually decreased, and the sperm motility and total sperm count also significantly decreased continuously (Table 2) (*P* < 0.05 vs. the DM group).

### 3.2. Diabetes Destroys Normal Testicular Physiological Structure in Mice

**Figure 1:**
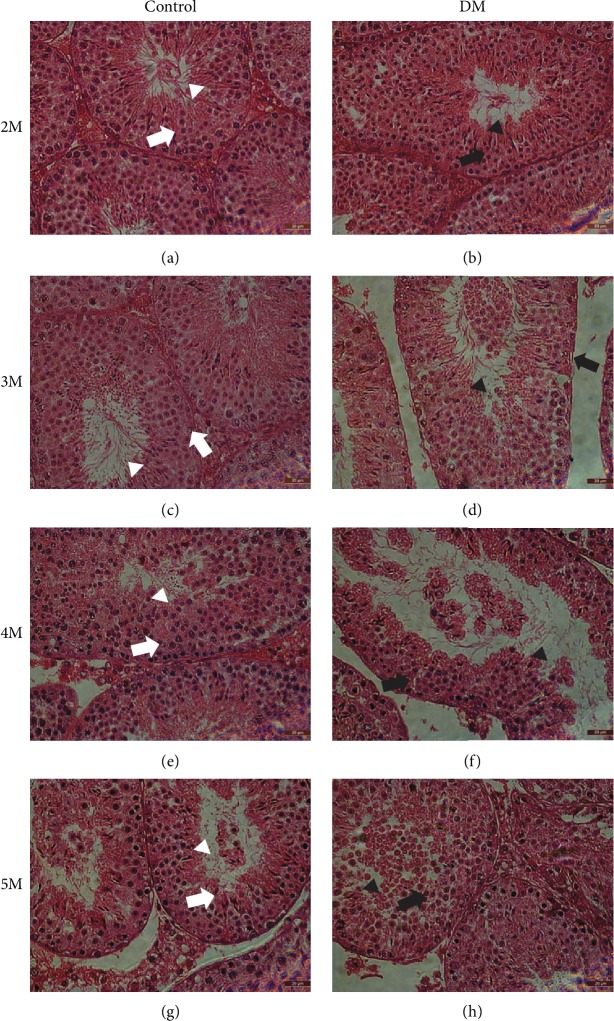
Histomorphological damage in diabetic mice. (a–h) HE staining of testes sections at 2 months, 3 months, 4 months, and 5 months. The white arrowhead shows a normal spermatogenic epithelium, the white triangle shows spermatogenesis, the black arrowhead shows fewer spermatogenic cell layers in the DM group than in the control group, and the black triangle shows sparse spermatogenesis in the DM group. Magnification = 400x; scale bar = 20 *μ*m; *n* = 4–5.

The physiological changes in the mice are shown in Figure 1. In the control group, spermatogenic cells at all levels of the spermatogenic epithelium were arranged in an orderly manner, as most of the spermatogenic epithelia had 6-7 layers, and numerous spermatozoa were found in the lumen of the seminiferous tubule. In contrast, the atrophy of and damage to the seminiferous tubules in the testes of diabetic mice resulted in a marked decrease in the number of cell layers in the seminiferous epithelia, with most having 3-4 or fewer layers, and spermatogenesis was significantly reduced in the seminiferous tubules when spermatogenic cells were lost. As the disease progressed, the damage gradually worsened. Within five months, the basic morphology of the testicular seminiferous tubules of the diabetic mice was completely destroyed and normal reproductive function was completely lost.

### 3.3. Expression of PK2/PKRs in the Testicular Tissue of Diabetic Mice

**Figure 2:**
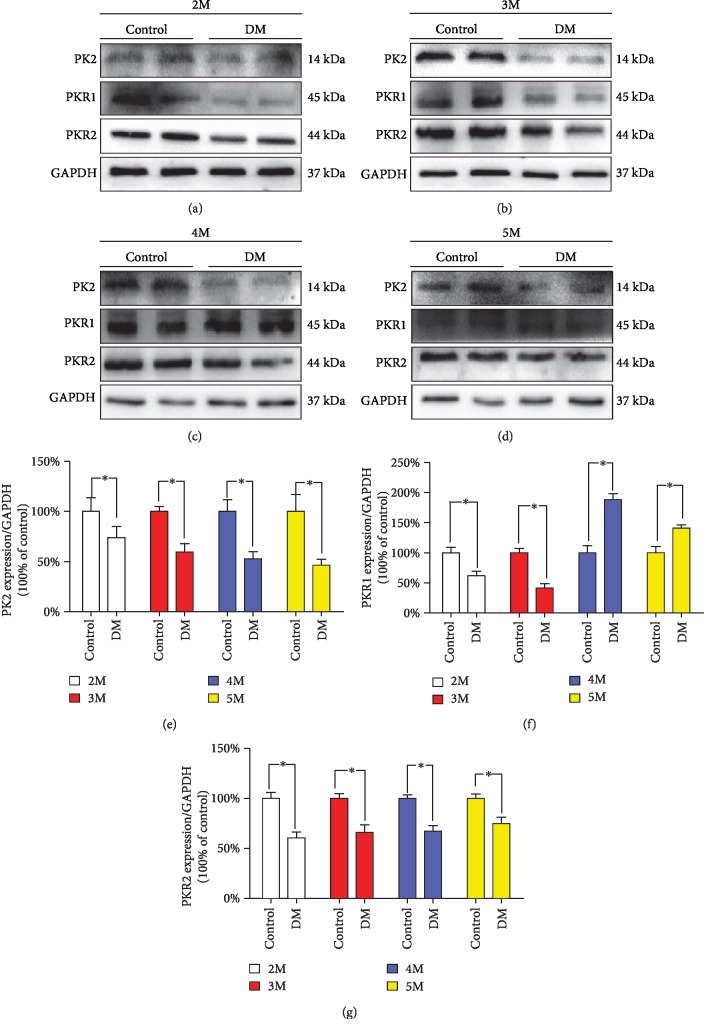
Testicular expression of the PK2/PKR proteins in the diabetic mouse growth process. (a) Typical protein expression of PK2, PKR1, and PKR2 at 2 months. (b) Typical protein expression of PK2, PKR1, and PKR2 at 3 months. (c) Typical protein expression of PK2, PKR1, and PKR2 at 4 months. (d) Typical protein expression of PK2, PKR1, and PKR2 at 5 months. (e) Quantification of PK2 protein expression at different time points. (f) Quantification of PKR1 protein expression at different time points. (g) Quantification of PKR2 protein expression at different time points. *n* = 4–6 per group. The values are expressed as the mean ± SEM. ^∗^*P* < 0.05 vs. the control group.

**Figure 3:**
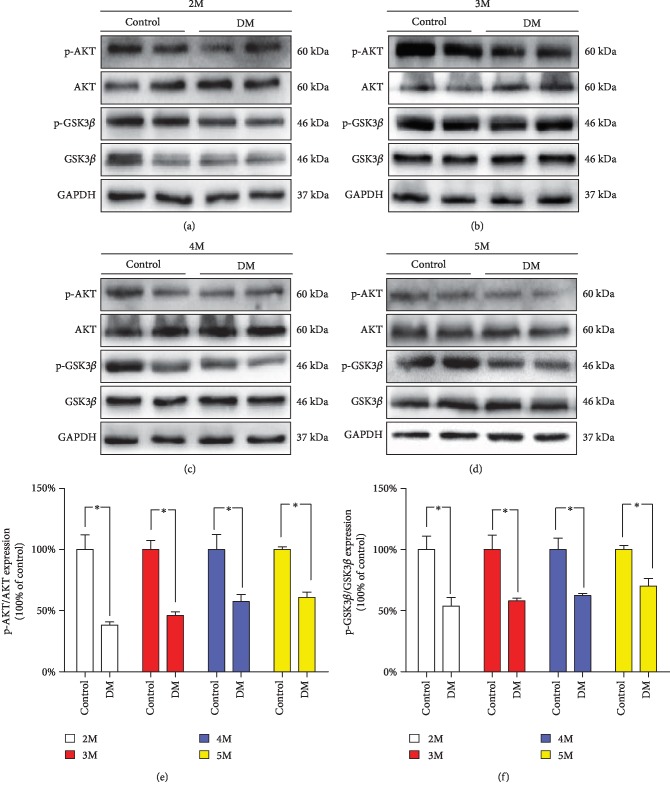
Testicular expression of the AKT/GSK3*β* proteins during the diabetic mouse growth process. (a) Typical protein expression of p-AKT, AKT, p-GSK3*β*, and GSK3*β* at 2 months. (b) Typical protein expression of p-AKT, AKT, p-GSK3*β*, and GSK3*β* at 3 months. (c) Typical protein expression of p-AKT, AKT, p-GSK3*β*, and GSK3*β* at 4 months. (d) Typical protein expression of p-AKT, AKT, p-GSK3*β*, and GSK3*β* at 5 months. (e) Quantification of p-AKT/AKT expression at different time points. (f) Quantification of p-GSK3*β*/GSK3*β* expression at different time points. *n* = 4–6 per group. The values are presented as the mean ± SEM. ^∗^*P* < 0.05 vs. the control group.

In previous reports, PK2/PKR2 were found to be important regulatory proteins for the normal physiological function of testicular tissue in mice. Therefore, we speculated that the PK2/PKR signalling pathway affects diabetic mice. As shown in Figure 2, the expression of PK2 and PKR2 was downregulated in diabetic mice compared with control mice. PKR1 levels were downregulated at 2 and 3 months and upregulated at 4 and 5 months in diabetic mice (Figure 2). AKT/GSK3*β* is the downstream target of PK2/PKRs. In our study, when the PK2/PKR2 protein expression was inhibited, AKT/GSK3*β* was also expressed at lower levels (Figure 3).

### 3.4. Expression of AKT/GSK3*β* in the Testicular Tissues of Diabetic Mice

It is known that the AKT/GSK3*β* pathway is associated with glycogen synthesis, cell growth, and survival. To further elucidate the potential mechanism underlying diabetes-induced testicular damage, we determined the effects of the AKT/GSK3*β* signalling pathway on testicular damage. As shown in Figure 3, a significant decrease in the phosphorylation of AKT and GSK3*β* was found in testis tissue of the DM group compared with the control group (Figure 3).

### 3.5. Met Improves the General State and Reproductive Capacity of Diabetic Mice

To improve the quality of life and reproductive ability of diabetic mice, we treated diabetic mice with Met for four months. After treatment, the hair of the diabetic mice gradually regained its lustre, the body weights of the mice increased, and the blood glucose levels were also well controlled (Table 3). Furthermore, the sperm vitalities and sperm numbers of the diabetic mice were increased significantly, which enhanced their reproductive potential (Table 3). Compared with the DM mice, the mice treated with Met had better reproductive potential. There were significant differences between the two groups (*P* < 0.05).

### 3.6. Met Improves Histomorphological Damage to Testicular Tissue in Diabetic Mice

**Figure 4:**
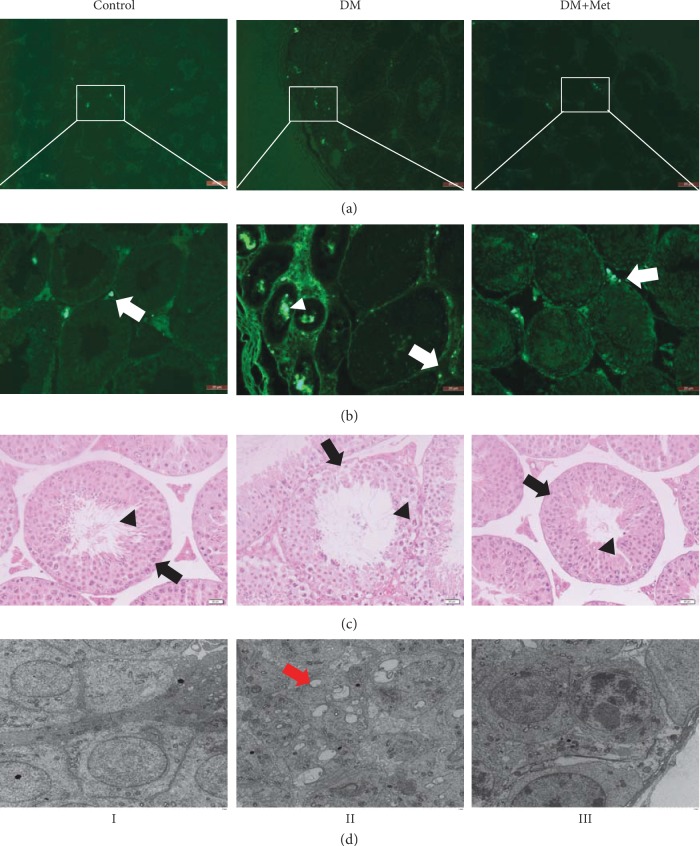
Histomorphological damage in diabetic mice. (a, b) Met prevented biotin from entering the seminiferous tubules. The white arrow indicates biotin between the tubules, and the white triangle indicates biotin in the seminiferous tubules. (a) Magnification = 100x; (b) magnification = 200x; scale bar = 20 *μ*m; *n* = 5. (c) Met reversed the morphology of testicular tissue in mice, as determined by HE staining. The black arrowhead shows the spermatogenic cell layers, and the black triangle shows spermatogenesis. Magnification = 400x; scale bar = 20 *μ*m; *n* = 5. (d) Met restored the organelle damage in testicular cells, and the red arrow indicates vacuolar degeneration or disintegration in the cytoplasm.

Similarly, in mice treated with Met, the morphology of the seminiferous tubules in the testes was similar to that in the control group. In the Met treatment group, the damage to the spermatogenic epithelium was significantly alleviated, the number of spermatogenic cell layers was noticeably increased, the cell arrangement was neat, and spermatogenesis was observed (Figure 4(c)). In the biotin tracer study, EZ-link Sulfo-NHS-LC-Biotin was distributed outside of the seminiferous tubules in the control group, but in the diabetic mice, biotin crossed the Sertoli cell gap. Met was able to repair the blood-testis barrier and prevent this damage (Figure 4(b)).

Transmission electron microscopy elucidated that the spermatogenic cells were orderly arranged at all levels of testicular tissue in the control group (Figure 4(d), I). In the DM group, the spermatogenic cells exhibited a disordered arrangement, and vacuolar degeneration or disintegration in the cytoplasm, unclear nucleoli in the nucleus, obvious mitochondrial oedema, massive chromatin aggregation, and widespread breakdown of intercellular bridges were observed (Figure 4(d), II). However, Met treatment normalized the alterations in the mitochondria, which exhibited slight oedema, and chromatin still accumulated (Figure 4(d), III).

### 3.7. Met Prevents Diabetes Mellitus-Induced Testicular Apoptosis

**Figure 5:**
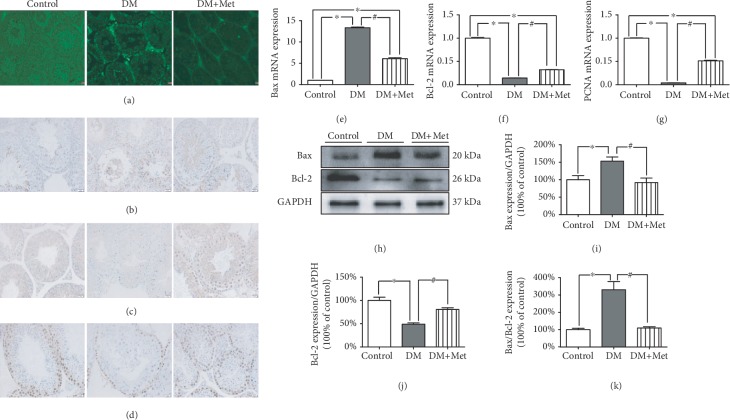
Met reduced diabetes mellitus-induced apoptosis in the testes. (a) A typical image of apoptotic cells stained by TUNEL (magnification = 100x; scale bar = 20 *μ*m). (b) Typical immunohistochemical staining of Bax (magnification = 200x; scale bar = 20 *μ*m). (c) Typical immunohistochemical staining of Bcl-2 (magnification = 200x; scale bar = 20 *μ*m). (d) Typical immunohistochemical staining of PCNA (magnification = 200x; scale bar = 20 *μ*m). (e) Quantification of Bax mRNA expression. (f) Quantification of Bcl-2 mRNA expression. (g) Quantification of PCNA mRNA expression. (h) A typical image of Bax and Bcl-2 proteins. (i) Quantification of Bax expression. (j) Quantification of Bcl-2 expression. (k) Quantification of the ratio of Bax/Bcl-2 expression. *n* = 3–6 per group. The values are presented as the mean ± SEM. ^∗^*P* < 0.05 vs. the control group; ^#^*P* < 0.05 vs. the DM group.

A TUNEL assay kit was used to detect the number of TUNEL-positive cells. The number of apoptotic cells was elevated in DM mice compared to control mice. Compared with untreated diabetic mice, diabetic mice treated with Met exhibited significantly inhibited diabetes mellitus-induced testicular apoptosis (Figure 5(a)).

It is well known that Bax and Bcl-2 are regulatory markers of apoptosis. Proliferating cell nuclear antigen (PCNA) is a sliding clamp for the DNA polymerase complex. In addition to having roles in DNA repair and DNA methylation, PCNA has been implicated in apoptosis. As demonstrated in Figure 5, the expression of Bcl-2 and PCNA was decreased, but Bax expression was increased in the testes of the DM group. Met treatment markedly reduced the ratio of Bax/Bcl-2 and increased PCNA expression in diabetic mice (Figure 5).

### 3.8. Met Activates Autophagy in Testicular Cells in Diabetic Mice

**Figure 6:**
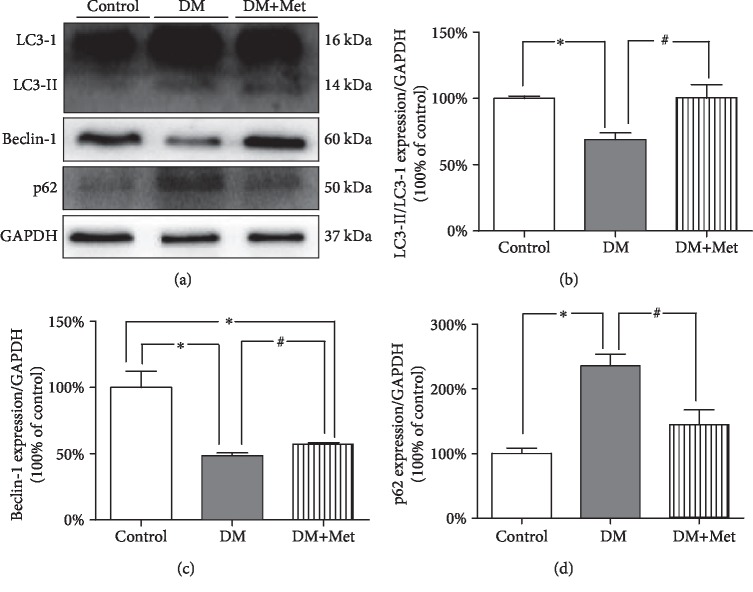
Met activated autophagy in the testes. (a) Typical protein expression of LC3B, Beclin-1, and p62. GAPDH served as the loading control. (b) Quantification of LC3B protein expression. (c) Quantification of Beclin-1 protein expression. (d) Quantification of p62 protein expression. *n* = 4 per group. The values are presented as the mean ± SEM. ^∗^*P* < 0.05 vs. the control group; ^#^*P* < 0.05 vs. the DM group.

To evaluate the effect of autophagy in testicular injury in diabetes mellitus, we selected the autophagy-related proteins Beclin-1, p62, and light chain 3B (LC3B) to observe changes in autophagy levels in diabetic mice after Met treatment. The data showed that Met effectively activated autophagy in the testicular tissue of mice (Figure 6).

### 3.9. Met Activates the PK2/PKR2 Pathway in Mouse Testes

**Figure 7:**
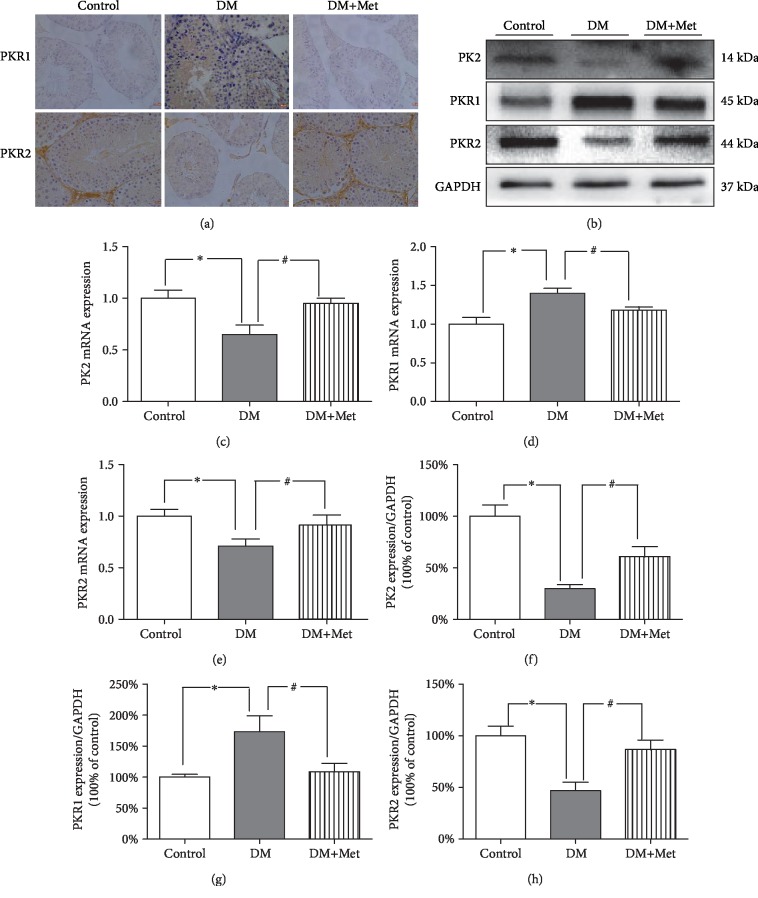
Met restores the protein expression of PK2/PKRs. (a) Typical immunohistochemical staining of PKRs (magnification = 400x; scale bar = 20 *μ*m). (b) Typical expression of PK2 and PKRs. (c) Quantitative analysis of PK2 expression. (d) Quantitative analysis of PKR1 mRNA expression. (e) Quantitative analysis of PKR2 mRNA expression. (f) Quantification of PK2 protein expression. (g) Quantification of PKR1 protein expression. (h) Quantification of PKR2 protein expression. *n* = 4–6 per group. The values are presented as the mean ± SEM. ^∗^*P* < 0.05 vs. the control group; ^#^*P* < 0.05 vs. the DM group.

In the above experiments, we proved that PK2/PKRs play an important role in the series of changes that occur during diabetic testicular injury. To evaluate the significance of Met, we analysed the levels of PK2/PKRs in testicular tissues by RT-qPCR, immunohistochemistry, and Western blotting. In contrast to those in the control group, the levels of PK2 and PKR2 were significantly decreased and the levels of PKR1 were increased in the testicular tissues of diabetic mice. Met supplementation significantly reversed these changes (Figure 7).

### 3.10. Met Activates the AKT/GSK3*β* Signalling Pathway

**Figure 8:**
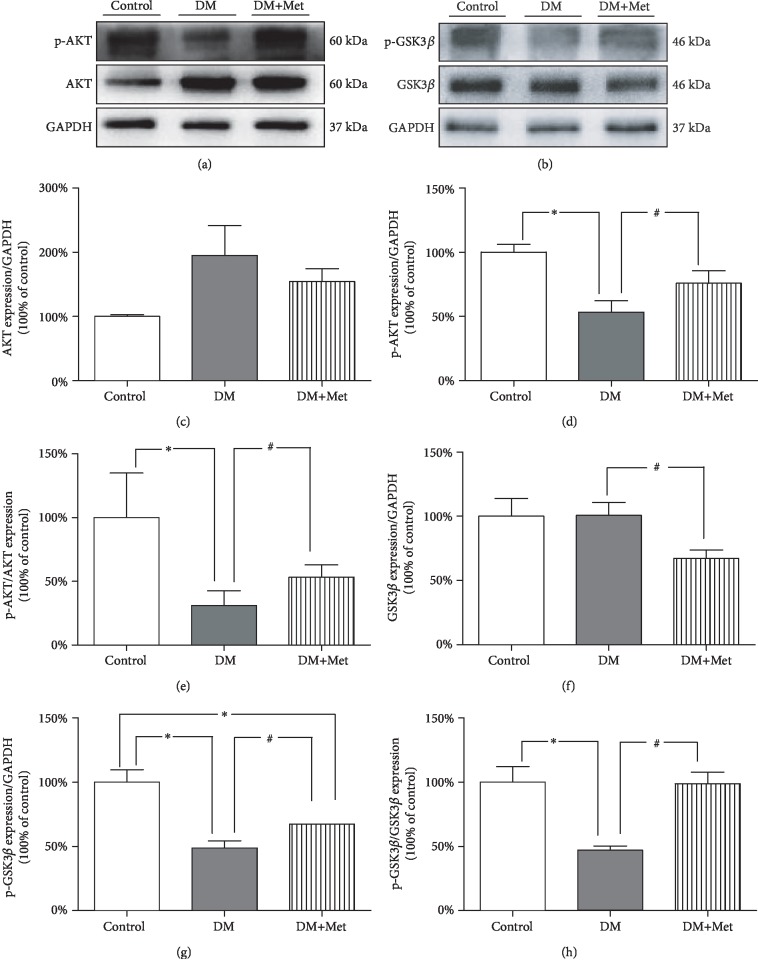
Met activates AKT/GSK3*β* protein expression. (a) Typical protein expression of p-AKT and AKT. (b) Typical protein expression of p-GSK3*β* and GSK3*β* proteins. (c) Quantification of AKT protein expression. (d) Quantification of p-AKT protein expression. (e) Quantification of p-AKT/AKT protein expression. (f) Quantification of GSK3*β* protein expression. (g) Quantification of p-GSK3*β* protein expression. (h) Quantification of p-GSK3*β*/GSK3*β* protein expression. *n* = 4–6 per group. The values are presented as the mean ± SEM. ^∗^*P* < 0.05 vs. the control group; ^#^*P* < 0.05 vs. the DM group.

Akt is a crucial downstream element of the PK2/PKR2 pathway, and the rapamycin receptor in mammals is a key regulator of autophagy under the mediation of AKT. Diabetes significantly decreased the phosphorylation of both AKT and GSK3*β* in the testis, a change that was reversed by Met (Figure 8).

## 4. Discussion

The findings of our study indicate that the PK2/PKR pathway plays an irreplaceable role in diabetic testicular injury and that Met protects against STZ-induced testicular impairment, apoptosis, and autophagy by regulating PK2/PKRs and restores the phosphorylation of the AKT/GSK3*β* signalling pathway in the testes. Apoptosis and autophagy are caused by glucose toxicity-induced testicular injury in patients with diabetes [15, 16]. While the clinical management of testicular injury remains challenging, our study suggests that Met can act as a treatment for testicular complications in diabetes. Our data demonstrate a likely role of PK2/PKRs in the beneficial effects of Met on diabetes-induced testicular injury.

It is well known that diabetes plays a crucial role in the pathology of testicular dysfunction by leading to the atrophy of the seminiferous tubules and damage to spermatogenetic cells, which are regarded as morphological indices of spermatogenesis dysfunction [17]. We found that diabetic mice exhibited noticeable morphological alterations in their testes, including damage to the structure of the seminiferous tubules and the blood-testis barrier, and a reduced sperm function. Upon treatment with Met, these changes were reversed in our study.

Similar to other GPCRs, the binding of PK2 and PKRs is regulated by local effects and/or endocrine hormones and is coupled with G proteins to mediate multiple biological actions [18]. Accumulating evidence has demonstrated that the PK2 protein in testicular tissue exists in only primary spermatocytes, and PK2 plays an essential role in inflammation by binding to PKR1, thus increasing the expression of PKR1. Interestingly, as a consequence of PK2 and PKR2 deficiencies, GnRH fails to be secreted, which results in dysfunctional sexual development and fertility in both male and female mice [12, 19–21]. To our knowledge, this is the first study to validate the changes in PK2 expression in the testes of mice with diabetes. Furthermore, our study focused on the presence of PKRs in mouse testes following STZ administration, which indicates that the expression of PK2 is expressed at low levels and in different patterns in the testes of diabetic mice. At the same time, PKR2 was noted to be overtly decreased and continuously expressed at a low level, while PKR1 was expressed at a lower level during the initial stage and then increased during the end stage of diabetes mellitus, which may be the main contributor to the expression of PK2 in response to inflammation and defective angiogenesis in diabetes. In addition, Met attenuated testicular injury by downregulating PKR1 activity, upregulating PK2/PKR2, and alleviating these pathologic alterations. The analysis indicates that the effect of Met is involved in regulating PK2 signalling pathways.

Cumulative evidence suggests that PK2/PKRs participate in myocardial survival, angiogenesis, and the haematopoietic system through the AKT and STAT3 signalling pathways [19, 22]. When culturing testicular tissue cells in vitro, the AKT signalling pathway regulates functional anchoring junctions, especially in Sertoli cells and spermatogenic cells [23]. Recent reports have indicated that higher AKT phosphorylation is tightly associated with testicular injury in STZ-induced diabetic mice; thus, we speculated that the AKT signal transduction pathway in testicular tissue could be used to investigate its effect on diabetes-induced testicular dysfunction. Our data suggest that the AKT/GSK3*β* signalling pathway continues to be inhibited during testicular injury in diabetes and that Met effectively reverses the diabetes-induced inactivation of AKT. It is conceivable that the uninterrupted high expression of PK2/PKR2 during Met treatment is beneficial for the activation of the AKT pathway.

Autophagy is a self-clearing process in eukaryotic cells. Normal autophagy can help maintain the balance of cell growth in organisms, especially to protect against testicular injury caused by hyperglycaemia and hypoxia [24]. However, abnormal autophagy is believed to be involved in the pathogenesis of many related diseases. Insufficient or excessive autophagy leads to the degeneration of germ cells. At the same time, it can degrade organelles, such as mitochondria and the endoplasmic reticulum, to destroy the stability of the testes, affecting their growth and development as well as their normal physiological functions [25]. Hyperglycaemia affects the mammalian target of rapamycin complex 1 (mTORC1) and leads to the inactivation of autophagy-related gene 4 (ATG4) by oxidation, ultimately leading to the lipidization of autophagy-related gene 8 (ATG8) and the formation of autophagy-related autophagosomes in the cell to induce autophagy [26]. The autophagy-related proteins Beclin-1, light chain 3B (LC3B), and ubiquitin-binding protein (p62/SQSTM1) are markers of autophagy activation in biological tissues. Beclin-1 and LC3B participate in the formation and elongation of autophagosomes, and p62, as a scaffold protein, binds ubiquitinated substrates and aids their aggregation and degradation by macroautophagy [27]. We noted suppressed autophagy, decreased Beclin-1 expression, a decreased LC3II-to-LC3I ratio, and increased p62 expression in diabetic mice, which corresponds to previous reports [28, 29]. Furthermore, we also found that Met treatment induces the enhancement of the autophagy level in testicular tissue.

Apoptosis, which is jointly regulated by the apoptosis-inducing gene Bax and the apoptosis-inhibiting gene Bcl-2, is considered a major factor in the possible mechanism of testicular injury induced by diabetes. An imbalance between Bax/Bcl-2 can activate the downstream caspase signalling pathway to induce apoptosis, which ultimately leads to spermatogenesis dysfunction [30]. Evidence suggests a link between autophagy and apoptosis pathways. Autophagy can remove excess cell components to inhibit apoptosis, and activation of the apoptosis-related protein caspase hinders the formation of autophagosomes [31]. Our study shows that the apoptosis of testicular tissue in diabetic mice is enhanced in parallel with increased Bax and decreased Bcl-2 and PCNA. When diabetic mice are treated with Met, the apoptosis of cells involved in the process of testicular injury is effectively inhibited. Therefore, our study supports a possible role for the PK2/PKR signalling cascade in the alteration of Met-elicited autophagic responses and the inhibition of apoptosis during diabetic toxicity.

In summary, our study strongly demonstrates that Met can regulate the PK2/PKR signalling pathway in testicular tissue, thereby eliciting AKT/GSK3*β* activity and regulating autophagic activity and apoptosis in testicular cells to protect the reproductive function of testicular tissue in mice. This study provides a new opportunity for the prevention and treatment of testicular reproductive injury, which is worthy of further clinical study.

## Figures and Tables

**Table 1:** Primer sequences for RT-qPCR.

Gene	Primer	Sequence
Bcl-2	Forward	5′-TGACTTCTCTCGTCGCTACCGT-3′
	Reverse	5′-CCTGAAGAGTTCCTCCACCACC-3′

Bax	Forward	5′-GCCTTTTTGCTACAGGGTTTCAT-3′
	Reverse	5′-TATTGCTGTCCAGTTCATCTCCA-3′

PCNA	Forward	5′-GTCGGGTGAATTTGCACGTA-3′
	Reverse	5′-CTCTATGGTTACCGCCTCCTC-3′

PK2	Forward	5′-CTCTATGGTTACCGCCTCCTC-3′
	Reverse	5′-GAAAGAAGTCCTTAAACACGCCA-3′

PKR1	Forward	5′-GAAAGAAGTCCTTAAACACGCCA-3′
	Reverse	5′-GACAGTCACAAAGCAGAGCGTA-3′

PKR2	Forward	5′-CTACTTCCTCTTCGTCTTCGGG-3′
	Reverse	5′-AGAAGTCTCGCACTATGGTAAAGC-′

*β*-Actin	Forward	5′-GTGACGTTGACATCCGTAAAGA-3′
	Reverse	5′-GTAACAGTCCGCCTAGAAGCAC-3′

**Table 2:** Metabolic abnormalities and reproductive capacity in diabetic mice.

Group	TW (mg)	BW (g)	TW/BW (mg/g)	Blood glucose (mmol/L)	Sperm vitality (%)	Sperm count (∗10^9^/L)
2M control	173 ± 5	29.2 ± 1.0	6.0 ± 0.3	7.2 ± 0.3	61.7 ± 4	1.86 ± 0.07
2M DM	131 ± 7^∗^	17.3 ± 0.4^∗^	7.7 ± 0.5^∗^	27.4 ± 0.2^∗^	63.4 ± 6	0.70 ± 0.10^∗^
3M control	185 ± 4	30.7 ± 0.4	6.0 ± 0.1	6.7 ± 0.6	51.4 ± 2	1.86 ± 0.13
3M DM	158 ± 5^∗^	21.3 ± 0.5^∗^	7.4 ± 0.2^∗^	27.3 ± 0.3^∗^	33.6 ± 3^∗^	0.51 ± 0.06^∗^
4M control	195 ± 3	30.8 ± 0.4	6.3 ± 0.1	6.2 ± 0.4	51.6 ± 5	1.51 ± 0.06
4M DM	162 ± 4^∗^	20.0 ± 0.4^∗^	8.1 ± 0.2^∗^	26.0 ± 1.3^∗^	27.8 ± 2^∗^	0.28 ± 0.08^∗^
5M control	183 ± 5	31.0 ± 0.4	5.5 ± 0.4	7.0 ± 0.2	47.2 ± 2	1.46 ± 0.07
5M DM	167 ± 4^∗^	21.6 ± 0.4^∗^	7.8 ± 0.2^∗^	26.0 ± 0.4^∗^	25.4 ± 2^∗^	0.27 ± 0.03^∗^

TW: testis weight; BW: body weight; TW/BW: testis weight/body weight. The data are the means ± SEM; ^∗^*P* < 0.05 vs. the control group; *n* = 9–15.

**Table 3:** Met prevents metabolic abnormalities and maintains reproductive capacity.

Group	TW (mg)	BW (g)	TW/BW (mg/g)	Blood glucose (mmol/L)	Sperm vitality (%)	Sperm count (∗10^9^/L)
Control	197 ± 7	31.6 ± 0.6	6.2 ± 0.2	6.3 ± 0.3	53.6 ± 2.0	1.75 ± 0.02
DM	158 ± 6^∗^	19.3 ± 0.5^∗^	8.2 ± 0.3^∗^	24.1 ± 1.4^∗^	26.7 ± 1.4^∗^	0.30 ± 0.06^∗^
DM+Met	173 ± 9	28.0 ± 0.4^∗^^#^	6.2 ± 0.3^#^	17.1 ± 1.6^∗^^#^	43.1 ± 4.6^∗^^#^	0.54 ± 0.05^∗^^#^

TW: testis weight; BW: body weight; TW/BW: testis weight/body weight. The data are the means ± SEM; ^∗^*P* < 0.05 vs. the control group; ^#^*P* < 0.05 vs. the DM group; *n* = 10–15.

## Data Availability

The data used to support the findings of this study are available from the corresponding author upon request.
